# Disrespect and abuse during facility‐based childbirth in central Ethiopia

**DOI:** 10.1080/16549716.2021.1923327

**Published:** 2021-08-17

**Authors:** Yohannes Mehretie Adinew, Helen Hall, Amy Marshall, Janet Kelly

**Affiliations:** a Adelaide Nursing School, The University of Adelaide, Adelaide, Australia; b College of Health Sciences and Medicine, Wolaita Sodo University, Sodo, Ethiopia; c Monash Nursing and Midwifery, Monash University, Melbourne, Australia

**Keywords:** human rights abuses, respect, birthing centers, Ethiopia

## Abstract

**Background:**

Respectful maternity care is a fundamental human right, and an important component of quality maternity care.

**Objective:**

The aim of this study was to quantify the frequency and categories of D&A and identify factors associated with reporting D&A among women in north Showa zone of Ethiopia.

**Method:**

A cross-sectional study was conducted with 435 randomly selected women who had given birth at public health facility within the previous 12 months in North Showa zone of Ethiopia. A digital (tablet-based) structured and researcher administered tool was used for data collection. Frequencies of D&A items organised around the Bowser and Hill categories of D&A and presented in the White Ribbon Alliance’s Universal Rights of Childbearing Women Framework were calculated. Multivariable logistic regression was used to identify the association between experience of disrespect and abuse and interpersonal and structural factors at p-value <0.05 and odds ratio values with 95% conﬁdence interval.

**Results:**

All participants reported at least one form of disrespect and abuse during childbirth. Types of disrespect and abuse experienced by participants were physical abuse 435 (100%), non-consented care 423 (97.2%), non-confidential care 288 (66.2%), abandonment/neglect (34.7%), non-dignified care 126 (29%), discriminatory care 99 (22.8%) and detention 24 (5.5%). Hospital birth [AOR: 3.04, 95% CI: 1.75, 5.27], rural residence [AOR: 1.44, 95% CI: 0.76, 2.71], monthly household income less than 1,644 Birr (USD 57) [AOR: 2.26, 95% CI: 1.20, 4.26], being attended by female providers [AOR: 1.74, 95% CI: 1.06, 2.86] and midwifery nurses [AOR: 2.23, 95% CI: 1.13, 4.39] showed positive association with experience of disrespect and abuse.

**Conclusion:**

Hospital birth showed consistent association with all forms of disrespect and abuse. Expanding the size and skill mix of professionals in the hospitals, sensitizing providers consequences of disrespect and abuse could promote dignified and respectful care.

## Background

While motherhood is often considered a fulfilling positive experience, pregnancy and childbirth-related complications are a leading cause of death for women of child-bearing age in developing countries [1]. Developing countries contribute 94% of global maternal deaths and more than half of these deaths occur in sub-Saharan Africa [2]. Ethiopia’s maternal mortality ratio, 401 per 100,000 live births in 2017, is one of the highest globally [3]. Birthing outside of a health facility without a skilled birth attendants present is the major reason behind this loss of life in Ethiopia [4,5].

Ethiopia aims to reduce its maternal mortality ratio to less than 70 per 100,000 live births by 2030 in order to achieve the United Nations Sustainable Development Goal 3 [6]. The Sustainable Development Goals are the blueprint to achieve a better and more sustainable future for all. Sustainable Development Goal 3 is about ‘Good Health and Well-being’ and is one of the 17 Sustainable Development Goals established by the United Nations in 2015. Ensuring access to quality obstetric care is essential as it has the potential to reduce up to 75% of preventable deaths [7,8]. The proportion of Ethiopian women who report having difficulty accessing health care decreased from 96% in 2005, to 70% in 2016 [9]. However, only 48% of women gave birth at health facility in 2019. Improving respectful maternity care has been flagged as a potential strategy for reducing preventable maternal mortality and morbidity, and to accelerate progress towards meeting the Sustainable Development Goal targets for improving maternal health [10].

Respectful maternity care is a key element of quality maternity care [11]. It is an approach that stresses positive interpersonal interactions between providers and women, throughout maternity care [12]. Women’s right to respectful and dignified health care throughout pregnancy and childbirth has become a central focus in intervention strategies that seek to reduce maternal mortality [13]; disrespect and abuse (D&A) affects women’s trust in care providers and the health system deterring them from seeking and using maternity care [12]. This highlights the need for maternity care to be competent and respectful if women are to use it [11].

Disrespectful treatment ranges from denial of a woman’s right to make informed decisions and being scolded for demanding their rights [14], to denial of anaesthesia while performing and repairing episiotomies [15]. The White Ribbon Alliance categorise D&A in childbirth into seven categories: physical abuse, non-dignified care, non-consented care, non-confidential care, abandonment, discrimination, and detention. Each category has more than one verification criteria with ‘Yes’ or ‘No’ dichotomized responses. According to White Ribbon Alliance, verification criteria/manifestations of D&A often fall into more than one category, so that categories are not intended to be mutually exclusive. Rather categories should be seen to be overlapping along a continuum [16]. International human rights frameworks highlight D&A of women during childbirth as a key human rights issue [17–20], and a human rights based approach to birthing care has become a primary concern [21].

Research from different parts of Ethiopia has identified growing evidence of disrespectful and abusive care during labour and childbirth regardless of women’s socio-demographic characteristics, such as age and level of education. However, some groups of women, such as unmarried women, women from low economic status and women with HIV sero positive status are more vulnerable to D&A based on these specific attributes [22–34]. Comprehensive information regarding D&A of women during childbirth is needed in order to more effectively implement policies, change practice, and culture and improve standards of care [35]. However, the experience of D&A and its determinants are not well understood. Thus, the aim of this study is to quantify the frequency and categories of D&A during facility-based childbirth and identify factors associated with reporting D&A among women in north Showa zone of Ethiopia.

## Methods

### Study design and setting

This study was conducted as part of a larger mixed methods study that examined disrespect and abuse of women during facility-based childbirth in Ethiopia. A community-based cross-sectional study (conducted in community settings rather than in a facility or institution) was conducted from 5 October 2019 to 25 January 2020 in North Showa zone of Ethiopia. The zone is located 110 km to north of the capital Addis Ababa. Based on the 2007 Census conducted by the Central Statistical Agency of Ethiopia, the Zone has a projected total population of 1.5 million in 2016, of whom 48% were women. North Showa zone has an area of 10,322.48 square kilometers and population density of 138.66 [36]. Three hospitals, 62 health centers, 268 health posts are currently functioning in the zone. Health posts were not included in the study as they do not provide birthing care. Women from across all districts (rural and urban areas) of the North Showa zone were included via a registry held by health extension workers.

### Study population and eligibility

Participants were women who had given birth at public health facilities of North Showa zone during the last 12 months preceding the survey, regardless of the birth outcome. Women who gave birth at home, those who were acutely unwell physically or mentally and those with disability which would prevent them talking to a researcher were excluded.

### Study variables

The dependent variables of this research were the seven categories of D&A women experience during facility-based childbirth (physical abuse, non-dignified care, non-consented care, non-confidential care, abandonment, discrimination, and detention) organised around the Bowser and Hill categories of D&A and presented in the White Ribbon Alliance’s Universal Rights of Childbearing Women Framework [16]. The independent variables of this research were sociodemographic variables (age, residence, marital, education, occupation and monthly household income), obstetric history and experience of maternity care utilization (walking distance of health facility from home, total number of live births, number of birth at health facility, antenatal care checkups, antenatal and childbirth in same facility, type of health facility visited for birth (health center vs hospital), method of birth and number of babies in most recent childbirth, profession and sex of providers who attended the birth).

### Sample size determination and sampling procedures

A single population proportion formula was used to calculate the sample size with assumptions of 78.6% proportion of disrespect and abuse [22], 4% precision, 95% level of confidence and a 10% non-response rate, the final sample size was 443. A list, including contact details, of women who gave birth at public health facility is maintained by health extension workers. Using this list as a sampling frame, 443 eligible women were selected by computer generated random numbers in Excel spreadsheet (Microsoft Corporation, 2013). Selection and initial contact was made in person by the health extension workers and women were invited to choose time and place of survey.

All health extension workers involved in data collection are female, have attended a one-year formal pre-service training conducted by trainers [37] Through a collaboration between the Ministry of Health and the Ministry of Education. The training includes didactic and clinical training regarding (1) family health services, disease prevention and control, (3) hygiene and environmental sanitation, and (4) health education and communication [38]. The main responsibilities of HEWs include health promotion, disease prevention, and treatment of uncomplicated and non-severe illnesses, such as cases of malaria, pneumonia, diarrhea, and malnutrition in the community. HEWs split their time between health posts and community settings; they are not directly connected to any birthing facilities [39].

### Data collection tool and procedures

Data were collected using digital, tablet-based tools, Open Data Kit Collect. A validated tool was programmed and uploaded to tablets for the survey [22,40]. The tool consisted of three parts. The first part contained seven questions and was used to assess the sociodemographic characteristics of participants. The second part contained 10 questions focusing on the participants’ obstetric history and experience of maternity care utilisation, and the third part included the seven categories of disrespect and abuse along with the 48 verification criteria used to measure experience of D&A. The tool was designed in English and translated to the local language, Amharic, and then back to English by a third person to check for internal consistency. The Amharic version of the tool was piloted and used to collect data face to face. Participants were surveyed in their homes or another preferred location and were accompanied by person of their choice during the survey.

### Measurement

We measured D&A using a framework developed by Bowser and Hill [41]. The D&A were categorised into seven groups and prevalence was calculated for each specific category. Each category has more than one verification criteria with ‘Yes’ or ‘No’ dichotomised responses. A respondent was considered to have been disrespected and/or abused for the specific category if she reported ‘Yes’ to at least one of the verification criteria in that category [22].

### Data quality assurance

Digital, tablet-based data collection improves quality of the data as it allows predetermined options only. Health extension workers with prior experience of tablet-based survey implementation conducted the survey following 2 days of intensive training. The health extension workers also practiced collecting data using the tablets in order to familiarize themselves with the tool prior to data collection. The data collectors were all females to make sharing ideas easier, as the topic is sensitive. The principal investigator has closely supervised the data collection process.

### Data processing and analysis

The data were exported to SPSS Window version 21. Descriptive statistics were used to describe the study population in relation to relevant variables. To identify predictors of each D&A categories, bivariate logistic regressions with each potential covariate were conducted, and variables that have p-value of <0.2 were included in the final multivariable binary logistic regression models. Then, four multivariable logistic regression models (one model for each category of D&A, except for physical abuse, non-consented care, and detention), with a 95% confidence interval were fitted. Physical abuse and non-consented care were reported by too many participants, whereas the number of women who reported detention was too small. As a result, these three categories were excluded as we could not perform further statistical analyses. Adjusted odds ratios and their 95% confidence intervals were computed and statistical significance was declared at p-value of <0.05.

## Results

Out of the invited 443 women, 435 agreed to participate in the study yielding a response rate of 98.1%. The mean age of respondents was 28.65 (SD = ± 5.38) ranging from 18 to 46 years. Over two-thirds, 304 (69.9%), of respondents were urban dwellers. Most 378 (86.9%) of participants were married and 100 (23%) have attended tertiary level of education, and only 77 (17.7%) of the participants reported monthly household income ≥1,644 birr (Table 1).

**Table 1.** Sociodemographic characteristics of participants (N = 435)

Variables		Frequency	Percentage
Age in Years	18–19	14	3.2
	20–24	78	17.9
	25–29	179	41.1
	30–34	90	20.7
	35+	74	27.0
Residence	Urban	304	69.9
	Rural	131	30.1
Marital status	Single	25	5.7
	Married	378	86.9
	Divorced	24	5.5
	Widowed	8	1.8
Level of education	No formal education	105	24.1
	Primary education	124	28.5
	Secondary education	106	24.4
	Tertiary education	100	23.0
Occupational status	Housewife	201	46.2
	Government employee	99	22.8
	Private employee	113	26
	Farmer	22	5.1
Monthly household income	≥ 1,644	77	17.7
	Less than 1,644	358	82.3
Spouse occupation	Unemployed	16	3.7
	Private employee	137	31.5
	Farmer	45	10.3
	Government employee	180	41.4

*1 USD was equivalent to 28.85 ETH birr during the study period and then multiplied by poverty line income (1.9/day).

### Participants’ obstetric history and experience of maternity care utilisation

More than half of the participants, 248 (57.0%) walk between 30 to 60 minutes to reach the nearest health facility. The majority, 316 (72.6%), had prior experience of giving birth at a health facility. Most, 395 (90.8%), of the study participants had antenatal care follow-up for their recent pregnancy, of which, 345 (87.3%) gave birth in same facility where they received antenatal checkups. About half, 223 (51.3%) gave birth in health centres. Midwives/nurses attended 255 (58.6%) of the women during birth. Approximately half 217 (49.9%) of women participants reported they were attended by male care providers Table 2.

**Table 2.** Obstetric history of participants (N = 435)

Variables		Frequency	Percentage
Walking distance of health facility from home in minutes	<30	145	33.3
	30–60	248	57.0
	>60	42	9.7
Total number of live births	1–3	354	81.4
	≥4	81	18.6
Number of births at health facility	1	119	27.4
	≥2	316	72.6
Antenatal follow up for recent pregnancy	Yes	395	90.8
	No	40	9.2
Antenatal and childbirth in same facility for recent birth (N = 395)	Yes	345	87.3
	No	50	12.7
Type of health facility visited for birth	Health Centre	223	51.3
	Hospital	212	48.7
Method of birth for recent childbirth	Vaginal delivery	391	89.9
	Caesarean section	44	10.1
Number of babies in most recent childbirth	One baby (single)	405	93.1
	Two babies (twin)	30	6.9
Profession of provider who attended the birth	Doctor	99	22.8
	Midwifery nurse	255	58.6
	I do not know	81	18.6
Sex of provider who attended the birth	Male	217	49.9
	Female	218	50.1

### Forms of disrespect and abuse reported by participants

**Figure 1.**
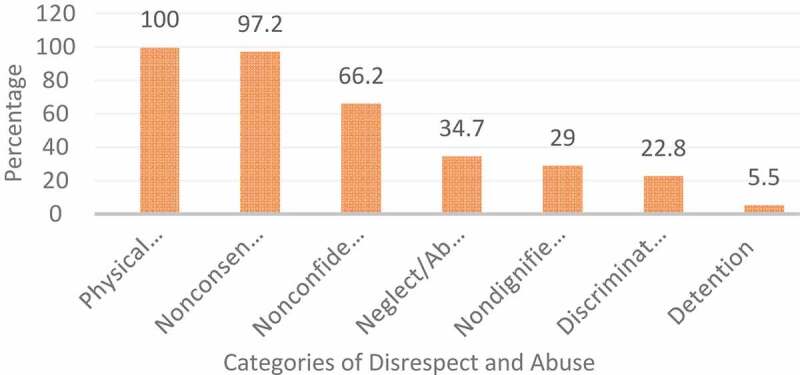
Categories of disrespect and abuse reported by women (N = 435)

Frequencies of D&A items organised around the Bowser and Hill categories of D&A and presented in the White Ribbon Alliance’s Universal Rights of Childbearing Women Framework were calculated. As a result, the prevalence self-reported D&A ranged from 100% for physical abuse to 5.5 for detention in health facility (Figure 1).

All women have reported at least one form of D&A during birth care. Physical abuse was found to be the most prevalent (100%) category. Among the verifications of physical abuse, 413 (94.9%) women reported that they were not allowed to give birth in their preferred birthing position; whereas for 381 (87.6%) women an episiotomy was given or sutured without anesthesia. Non-consented care was the second most common category of D&A reported by 423 (97.2%) of participants. A vast majority of participants, 362 (83.2%) reported that care providers had conducted a vaginal examination without their consent. In addition, 92 (21.1%) participants reported that intrauterine device was inserted without their consent. Two-thirds, 288 (66.2%) of participants reported confidentiality of their care was breached, of which 220 (50.6%) reported absence of curtains/partitions or other measures to provide privacy. Likewise, 204 (46.9%) of participating women said vaginal examinations were not conducted privately. About one-third (34.7%) of participants felt abandoned or neglected. More than one in ten (12.9%) women said providers were not present when the baby was born. Non-dignified care was the fifth most common D&A reported by 126 (29%) participants. Among verifications of non-dignified care, 75 (17.2%) of women said providers threatened them if they did not comply and indicated that they or their baby would have a poor outcome. Similarly, 71 (16.3%) of participating women reported that healthcare providers had shouted/screamed at them during labour or birth. Nearly quarter, 99 (22.8%) of participants reported that they felt discriminated against. Discrimination amongst this group included 51 (11.7%) respondents stating healthcare providers made negative comments regarding their HIV seropositive status, whereas 37 (8.5%) women received negative comments regarding their age. In addition, 24 (5.5%) of respondents said they were detained in the facility against their will, of which, 17 (3.9%) were detained due to inability to pay hospital bills, while 13 (3.0%) were instructed to clean up their own blood or other fluid after birth (Table 3).

**Table 3.** Experiences of disrespect and abuse during facility-based childbirth, Ethiopia, 2020 (N = 435)

Types of D&A	N	%
**Experienced at least one form of D&A**	**435**	**100**
**Physical abuse**	**435**	**100**
Pinched/kicked/slapped	34	7.8
Hit with an instrument	12	2.8
Gagged to prevent from speaking/ making noise	41	9.4
Restrained or tied down during labor	32	7.4
Fundal pressure applied	75	17.2
Preferred birthing position not allowed	413	94.9
Episiotomy given or sutured without anesthesia	381	87.6
Not allowed to move around during labour	277	63.7
Had no access to water or other oral fluids	108	24.8
Not allowed to eat without medical indication	219	50.3
**Non-dignified care**	**126**	**29**
Shouted/ screamed at	71	16.3
Insulted/ scolded/ mocked	44	10.1
Received negative comments about your/your baby’s physical appearance	37	8.5
Received negative comments regarding your sexual activity	32	7.4
Threatened with a physical violence or unfavorable medical procedures	59	13.6
Threatened with poor outcome to comply	75	17.2
Threatened to withhold care	55	12.6
Blamed or intimidated during childbirth	17	3.9
Hissed at	36	8.3
**Non-consented care**	**423**	**97.2**
Vaginal examination not explained	126	29.0
Non-consented vaginal examination	362	83.2
Vaginal examination not done privately	152	34.9
Non-consented episiotomy	58	13
IUD insertion	92	21.1
Augmentation of labor	97	22.3
**Non-confidential care**	**288**	**66.2**
Staff discussed private information in a way that others could hear	54	12.4
Lack/misuse of curtains to provide privacy	220	50.6
Do not have bed during labor	89	20.5
Do not have during childbirth	73	16.8
Shared bed with another woman or women	31	7.1
Vaginal examinations not conducted privately	204	46.9
**Neglect/ abandonment**	**151**	**34.7**
Providers not present when the baby came out	56	12.9
Felt ignored by the health workers or staff	111	25.5
Waited for long before attended by a provider	107	24.6
Felt emotionally unsupported by providers	64	14.7
Providers did not listen to my concerns	77	17.7
Providers did not respond to my concerns	84	19.3
Disallowed to have a birth companion	91	20.9
**Discrimination**	**99**	**22.8**
Received negative comments regarding your ethnicity/ religion	7	1.6
Received negative comments regarding your age	37	8.5
Received negative comments regarding your marital status	11	2.5
Received negative comments regarding your level of literacy	22	5.1
Received negative comments regarding your economic circumstance	24	5.5
Received negative comments regarding your HIV seropositive status	51	11.7
Denied a language interpreter	13	3
**Detention**	**24**	**5.5**
Detained in the hospital due to inability to pay hospital bills	17	3.9
Instructed to clean up own blood or other fluid after birth	13	3.0
Asked for a bribe, informal payment/ gift	11	2.5

### Predictors of disrespect and abuse

Four logistic regression models were constructed to examine the relationship of interpersonal and structural factors with each of the four different categories of D&A. Three forms (physical abuse, non-consented care and detention) were excluded since bivariate analysis revealed that further statistical analyses could not be performed due to the number of women who reported these categories being either too high or too small Table 4.

**Table 4.** Bivariate and multivariate logistic regression analysis output of factors associated with disrespect and abuse during facility-based childbirth, North Showa zone, Ethiopia, 2019

Variables with categories	Non-dignified care AOR [CI]	Non-confidential care AOR [CI]	Discriminatory care AOR [CI]	Abandonment AOR [CI]
**Level of education**				
Tertiary	1	1	1	1
No education	1.41 (0.61–3.26)	2.12 (0.96–4.67)	1.59 (0.72–3.51)	1.08 (0.51–2.27)
Primary	2.04 (0.90–4.62)	2.15 (1.01–4.55)*	0.89 (0.39–2.01)	1.15 (0.55–2.37)
Secondary	1.58 (0.69–3.65)	2.24 (1.09–4.60)*	1.60 (0.75–3.44)	1.42 (0.69–2.90)
**Residence**				
Urban	1	1	1	1
Rural	3.65 (2.11–6.32)*	1.44 (0.76–2.71)	1.07 (0.59–1.92)	1.77 (1.04–3.02)*
**Household monthly income**				
≥1644	1	1	1	1
Less than 1644	5.29 (1.94–14.42)*	2.26 (1.20–4.26)*	2.82 (1.11–7.11)*	1.73 (0.83–3.56)
**Type of health facility visited for birth care**				
Health center	1	1	1	1
Hospital	1.75 (1.04–2.94)*	3.04 (1.75–5.27)*	2.16 (1.24–3.77)*	2.58 (1.57–4.25)*
**Profession of provider attended birth**				
Doctor	1	1	1	1
Midwifery nurse	2.23 (1.13–4.39)*	3.43 (1.90–6.18)*	2.49 (1.14–5.46)*	0.87 (0.45–1.59)
I don’t know	1.95 (0.84–4.54)	4.00 (1.79–8.93)*	3.91 (1.59–9.58)*	0.50 (0.23–1.10)
**Sex of provider attended the birth**				
Male	1	1	1	1
Female	1.74 (1.06–2.86)*	4.21(2.52–7.03)*	0.78 (0.47–1.30)	4.0 4(2.51–6.50)*

*Significantly associated at p value of <0.05.

The type of health facility at which childbirth takes place, monthly household income, profession and sex of providers were found to be significantly associated with most of the D&A categories. The type of health facility at which childbirth takes place was found to be the most important statistically significant predictor of all forms of D&A; those who gave birth at hospitals were three times more likely to experience disrespect or abuse [AOR: 3.04, 95% CI: 1.75, 5.27] than those who gave birth at health centers. Experiences of disrespect or abuse were 1.4 times more likely to be reported by rural women than their urban counterparts [AOR: 1.44, 95% CI: 0.76, 2.71]. Women who have monthly household income less than 1,644 Birr (USD 57) were about two times more likely to experience disrespect or abuse [AOR: 2.26, 95% CI: 1.20, 4.26]. Regarding sex and profession of care providers, those women who were attended by female providers [AOR: 1.74, 95% CI: 1.06, 2.86] and midwives/nurses [AOR: 2.23, 95% CI: 1.13, 4.39] were more likely to report disrespect or abuse compared to those who were attended by male providers and doctors, respectively.

## Discussion

Disrespect and abuse during facility-based childbirth is a global problem with varying degrees of severity and differing drivers in different contexts [42]. It is often a greater problem in developing countries where inadequate number of care providers serve large proportion of clients [43]. There is growing evidence in Ethiopia that women are experiencing disrespect and abuse in birthing facilities [44,45]. Thus, this study sought to quantify the frequency and categories of D&A and identify factors associated with reporting D&A among women in north Showa zone of Ethiopia.

Respectful maternity care is a universal right of every childbearing women; however, this study reveals that D&A are common in health facilities. Every woman who participated in this study has experienced at least one form of D&A, and this rate is higher than for any other study. This disparity is attributed to the comprehensiveness of the current research which used 48 verification criteria for the seven categories of disrespect and abuse, whereas other studies used only 25 or less [22,24–26,28,32,34], which may have led to under reporting of disrespectful and abusive care. However, two studies from Arbaminch, Ethiopia and Enugu, Nigeria have reported almost similar rate 98.9% [33] and 98% [46] of D&A.

Physical abuse is the most prevailing category with 100% prevalence. From these, 413 (94.9%) women were not allowed to give birth in their preferred birthing position and for 381 (87.6%) women an episiotomy was given or sutured without anesthesia. Whereas 34 (7.8%) and 12 (2.8%) participants were pinched/kicked/slapped and hit with an instrument, respectively. Other research from Ethiopia reported level of physical abuse that ranged from 2% to 75.2% [22,24–26,29,31,32,34].

When interventions become necessary, service providers should provide the mother with sufficient information in a language she can comprehend so that she can knowingly refuse or consent to the intervention [47]. However, this study found that 97.2% of participating women reported non-consented care. This finding is higher than findings of previous research across all contexts [26,32,48]. Most, 362 (83.2%) of women who reported at least one vaginal examination also reported that they did not provide consent, and a significant proportion 152 (34 · 9%) reported that vaginal examinations were not conducted privately. A cross-sectional study conducted in four African countries with labour observations and community-based surveys reported that among women with at least one observed vaginal examination, at their first vaginal examination 847 (59 · 0%) did not provide consent, whereas 2611 (59 · 4%) vaginal examinations were done without consent across all women [48]. Best practice is that providers respect the privacy and confidentiality of every childbearing woman during counseling, physical examinations, and clinical procedures, as well as in the handling of patients’ medical records and other personal information. However, two-thirds of women who participated in this study had experienced a breach in confidentiality. Studies from other parts of the country have similarly revealed a high level of non-confidential care; common examples include the lack of privacy curtains and women not being appropriately covered during intimate examinations and/or labor and birth [22,24,32].

Every woman is a person of value and is worthy of respect. All words, actions, and non-verbal communication of providers must honor the dignity of each woman. Unfortunately, in this study, 29% of women reported non-dignified care. Previous studies have documented similar result elsewhere in Ethiopia [22,24,32,34]. Likewise, Firew at al. found non-dignified care in agreement with these findings that 31.4% were shouted at, 13.7% experienced threat of withholding treatment and 17.2% were blamed or intimidated [26]. Non-dignified care and insults may drive women away from healthcare facilities towards less trained providers who treat them with dignity and respect [46]. Service providers must acknowledge that women have the right to be treated with respect and consideration. In this study, however, a number of women reported negative comments regarding their HIV seropositive status, age and literacy were 11.7%, 8.5% and 5.1%, respectively.

Attentive care is the right of each client and a woman should never feel abandoned during labour or immediately after birth. However, our study demonstrated that 40% of participating women felt ignored/abandoned and 12.9% of women reported healthcare providers were not present when the baby was born. This finding is in line with a study conducted in the southwest of Ethiopia [32]. Furthermore, women should be able to have a birth companion of their choice. However, more than half of the women in this study reported that this was disallowed. The mere presence of a birth companion can ensure respectful care [49], whereas restricting the presence of a birth companion is reported to be a significant barrier to humanized birth care [26,50,51]. This suggests that healthcare providers know the way they behave in the absence of a companion is inappropriate and treat a client differently when a companion of the client is present [52].

Freedom from detention is the right of each childbearing woman and a woman or her baby or her companion should never be forcibly kept in a facility. Detention is the least-reported category by participants, and this is similar to rates reported in other studies from Ethiopia [22,24,26,30,32–34] possibly because maternity services are free of charge in Ethiopia and detention due to unaffordable service bills are rare. The economic status of women has been identified as a significant barrier to quality care. Unlike financially secure families, poor women are more likely to experience disrespectful birthing care [53]. Similarly, women of low economic status were more likely to experience D&A in the current research. This indicates the prevailing social attitudes towards people from lower socioeconomic backgrounds. Studies from different contexts have also revealed likewise [26,54].

Women who gave birth at hospitals reported higher level of D&A compared to those who gave birth at health centers. Hospitals generally belong to the secondary and tertiary levels in Ethiopian three-tier healthcare system. They serve as referral sites for health centers, which are primary level. Many women prefer hospitals over health centers as they are higher-level and are expected to give better medical care [27]. Increased client volume and insufficient staffing may impede the provision of respectful maternity care in hospitals [55]. Previous studies have identified that working in under-equipped and overwrought health systems affects provider enthusiasm and is often labelled as significant contributor to D&A in facilities [56]. Local studies have similarly reported higher incidence of D&A among women who gave birth at hospitals [22,25,26,31,33].

Another predictor for experience of D&A was type of professional who attended the birth. Some professions were reported to disrespect and abuse women than the others. Women who were attended by midwives/nurses were more likely to experience disrespect and abuse compared to those who were attended by doctors. A study from Nigeria revealed that midwives found more of the presented scenarios of mistreatment to be acceptable practices, compared to doctors [14]. Middle and lower level care providers, midwives/nurses, work the entire shift, whereas higher level (doctors) are mostly called to the labor ward to handle complications or cesarean delivery. Attending to the highly eventful and sensitive process of labor and delivery for long hours in poor working conditions may lead to providers burnout [57] and increase their likelihood of inappropriate treatment of mothers. Negative attitudes of providers towards women is attributed in part to being overworked [58]. Evidence shows that if lower level providers are the victims of D&A by managers or higher level providers, then it is more likely that they will use D&A as a tool to ascertain power [59].

Female care providers are presumed to treat women better. Unexpectedly, women who were attended by female providers had a greater likelihood of experiencing D&A in this study. Similar findings have been indicated in a study from Mozambique where higher odds of D&A were reported among women attended by female care providers [60]. On the other hand, rural residence was associated with increased likelihood of experiencing D&A. Similarly, in study from Mozambique, the occurrence of disrespect and abuse was much higher in the district hospitals compared to the central hospital [60].

## Limitation of the study

Only women who had given birth at a health facility were included. This possibly excluded women who might have chosen not to visit the health facility due to previous experiences of D&A.

## Conclusion

The level of disrespect and abuse reported by participants is high. The drivers and enablers include both structural and interpersonal factors. Hospital birth showed consistent association with all forms of disrespect and abuse. Expanding the size and skill mix of professionals in the hospitals, sensitizing providers and health managers regarding the magnitude and consequences of D&A could decrease the workload and possibly promote more dignified and respectful maternity care, respectively.

## Paper context

Disrespect and abuse of women during facility-based childbirth includes the way care providers act, behave or treat childbearing women and is against the basic human right of childbearing women. There are seven categories of disrespect and abuse and each category has its own verification criteria. No study known to the authors has been done on this issue in the study area so far. The authors surveyed mothers who had given birth at health facility to share their experience. The level of disrespect and abuse reported is high compared to previous studies.
